# Local and Global Visual Processing in 3-Year-Olds With and Without Autism

**DOI:** 10.1007/s10803-018-3470-8

**Published:** 2018-02-06

**Authors:** Elisabeth Nilsson Jobs, Terje Falck-Ytter, Sven Bölte

**Affiliations:** 1 Uppsala Child and Baby Lab, Department of Psychology, Uppsala University, 751 42 Uppsala, Sweden; 2 Division of Neuropsychiatry, Department of Women’s & Children’s Health, Center of Neurodevelopmental Disorders (KIND), Karolinska Institutet, Stockholm, Sweden; 3 Center of Psychiatry Research, Stockholm County Council, Stockholm, Sweden; 4 Child and Adolescent Psychiatry, Stockholm County Council, Stockholm, Sweden

**Keywords:** Autism, Neurodevelopmental disorder, Cognition, Visual perception, Local processing, Child development

## Abstract

Research on visual local and global perception in Autism Spectrum Disorder (ASD) is incomplete in young children. We investigated 35 three-year-old siblings of children with ASD, either diagnosed (*n* = 12) or not diagnosed (*n* = 23) with ASD as well as 14 controls with typical development and with no family history of ASD. Data from the local tasks Children’s Embedded Figures Test, Hidden Pictures, Figure-Ground and the global tasks Closure and Fragmented Picture Test were collected. Enhanced performance on the local task Hidden Pictures differentiated children with ASD from the other groups. Implications of these results are discussed.

**Electronic supplementary material:**

The online version of this article (10.1007/s10803-018-3470-8) contains supplementary material, which is available to authorized users.

## Introduction

Several cognitive models of Autism Spectrum Disorder (ASD) suggest that attention to detail is related to the condition (Mottron et al. 2006; Plaisted 2001; Baron-Cohen 2002; Happé and Frith 2006). Detail focus is often referred to as local perception as opposite to global perception, i.e., focusing on the whole of e.g., a picture or integrating parts of a feature into a whole. Children with typical development (TD) can perceive both local and global features already at an early age (Stiles et al. 1991) and there are indications that individuals with TD develop a global visual default mode, perceiving global and local information as accurate when instructed to, but are faster at reporting global features in spontaneous answers (Campana et al. 2016). This global precedence has been found to remain in many individuals throughout life (Bruyer and Scailquin 2000) but results can vary depending on ethnicity (McKone et al. 2010) and method used (Dale and Arnell 2013). In individuals with ASD, on the other hand a visual *local* processing default mode has been found in spontaneous responses (Wang et al. 2007; Happé and Frith 2006) although processing global information in ASD appear as accurate as in TD when explicitly and accurately demanded (Koldewyn et al. 2013). A local default mode has also been found in individuals with elevated autistic traits (Stevenson et al. 2016). A common paradigm to examine local–global default modes is to use stimuli where both global and local information levels are present, as they allow to investigate the trade-off effects in the perception and interference of one stimulus level in the presence of another. Findings of local-to-global and global-to-local interference in individuals with ASD or autistic traits have been mixed (Plaisted et al. 1999, 2006; Rinehart et al. 2000; Nayar et al. 2017; Deruelle et al. 2006; Stevenson et al. 2016; Van der Hallen et al. 2015, 2017).

Another approach is to examine local and global processing skills separately. Given evidence that local processing matures before global processing during typical development (Guy et al. 2016), and the indications that local and global processes are independent (Porporino et al. 2004), it appears reasonable to study these functions separately in ASD and TD, particularly in young children (Happé and Booth 2008). Local figure-ground tests (e.g., Shah and Frith 1983; Witkin et al. 1971) and visual search tasks have been quite common with results showing faster reaction times or higher accuracy in children with ASD in comparison to children with TD (Morgan et al. 2003; Kaldy et al. 2011; Pellicano 2006; Happé and Frith 2006; O’Riordan and Plaisted 2001) and positive correlations between autistic symptoms and local performance (Gliga et al. 2015; Cheung et al. 2016; Koldewyn et al. 2013; van Eylen et al. 2015). However, other studies find no group differences or inconsistent results (White and Saldana 2011; Muth et al. 2014; Horlin et al. 2016). Regarding research on global performance, tasks vary considerably, tapping into both visual and other cognitive abilities such as matching (Olu-Lafe 2015), drawing (Booth et al. 2003), puzzle-like tasks or deduction of the whole object from a part (Jolliffe and Baron-Cohen 2001; Nakano et al. 2010). A few studies have used visual global object/animate integration tasks in line with the definition of visual closure according to the Cattell-Horn-Carroll (CHC) factorial model of cognitive abilities (Schneider and McGrew 2012; Flanagan and Dixon 2013, p. 8). Here, tasks such as fragmented pictures (Snodgrass and Corwin 1988; Kessler et al. 1993) are used, i.e., animates or objects presented in a fragmented, shattered way that are to be recognised. Using fragmented stimuli is of particular interest when investigating children as possible confounds, such as of motor skills and deduction are controlled to a relatively high degree. Similar to results on local tasks, results differ from no difference between individuals with ASD and TD (Mottron et al. 2003) to global processing being slower or less accurate in individuals with ASD compared to TD (Booth and Happé 2016; Bölte et al. 2007; Scheurich et al. 2010) or mixed results (van Eylen et al. 2015). To the best of the authors knowledge, research including global performance, either as part of “trade-off” paradigms or real object/animate integration tasks in pre-school children with ASD has not been conducted.

In conclusion, the understanding of local and global processing in pre-school aged children with ASD is incomplete. Therefore, the objective of the current study was to examine local and global visual processing using separate measures for local and global perception, in 3-year-olds. An increasingly applied methodology to study early trajectories in ASD is high-risk (HR) for ASD sibling research (Bölte et al. 2013; Zwaigenbaum et al. 2015). HR-siblings are younger brothers or sisters to individuals diagnosed with ASD. Compared to about 1–2% in the general population (CDC 2016), the prevalence of ASD in siblings is about 14–20% (Messinger et al. 2013). In the current study we used cross sectional assessments of 3-year-olds that had been ascertained in a HR ASD longitudinal sibling design. We examined HR siblings with ASD (HR-ASD group), high-risk siblings without ASD (HR-noASD group) and low risk (LR) for ASD children, i.e., TD controls (LR group). We expected superior local performance in the HR-ASD group compared to the other groups. For global performance we tentatively expected superior performance in the HR-noASD and LR groups compared to the HR-ASD group.

## Methods

### Participants

The project was approved by the Regional Ethical Board in Stockholm. Participants were part of the longitudinal Early Autism SwEden (EASE; smasyskon.se) sibling project, including siblings with HR and LR for ASD. Diagnosis at 36 months, reliable at this age in most cases (Ozonoff et al. 2015), was based on consensus of two experienced clinicians according to DSM-5 criteria (American Psychiatric Association 2013), endorsed by information from the Autism Diagnostic Observation Schedule-2 (ADOS-2; Lord et al. 2012), module 1 or 2; the Autism Diagnostic Interview-Revised (ADI-R; Lord et al. 1994); the Vineland Adaptive Behavior Scales-2 (VABS-II; Sparrow et al. 2005; McDonald 2014; Mouga 2014) and the Mullen Scales of Early Learning (MSEL; Mullen 1995). Parents were also asked to complete a checklist about their child’s expressive vocabulary containing 22 words used in the global tasks requiring verbal answers. Subsequent to diagnostic assessment, the HR siblings were divided into those with ASD (HR-ASD group) and without ASD (HR-noASD group). One child in the LR group fulfilled criteria for ASD, and was excluded from the analysis. One child from each of the three groups was excluded from the final sample, due to a general inability to perform the local and global tasks. Thus, excluding four children, 49 three-year-old children were included in total (Table 1), 12 in the HR-ASD group (5 boys, 7 girls), 23 in the HR-noASD group (7 boys, 16 girls) and 14 in the LR group (6 boys, 8 girls). Groups were largely comparable for age, MSEL: verbal IQ and expressive vocabulary according to the parent checklist, but not MSEL, where there was a group difference for non-verbal IQ between the LR and the HR-ASD group. As expected, autistic symptoms and traits, differed between groups, the HR-ASD group had higher ADOS-comparison scores and lower VABS-II scores on the social domain than both HR-noASD and LR groups. Scores on the ADI-R differed between the LR group and both HR-noASD and HR-ASD groups, but not between the latter groups. Regarding other neurodevelopmental symptoms, in the HR-noASD group six children had signs of ADHD, two of speech and language impairment and one of developmental delay. In the HR-ASD group two children had signs of ADHD and three of receptive language. In the LR group there were no signs of other developmental concerns.

**Table 1:** Sample characteristics

	LR (*n* = 14)			HR-noASD (*n* = 23)			HR-ASD (*n* = 12)			Group comparisons		
	*Mean*	*SD*	*Min–Max*	*Mean*	*SD*	*Min–Max*	*Mean*	*SD*	*Min–Max*	*F*/F*	*p*	Post hoc
Age. months	37.65	2.34	36–45	39.39	4.25	36–49	40.09	4.33	36–48	1.55	0.228	*ns*
Mullen: VIQ	111.82	10.48	93–126	103.65	21.44	62–149	99.25	23.94	76–160	1.40	0.256	*ns*
SpecVoc: GC. raw sc^a^	17.36	0.93	15–18	16.05	2.28	10–18	16.45	1.37	14–18	3.08	0.057	*ns*
Mullen: NVIQ	116.96	10.42	101–135	112.96	14.81	80–139	103.58	13.12	80–124	3.44	0.041	HR-ASD > LR***
ADOS-2 Comparison sc	2.36	1.60	1–6	2.91	1.35	1–6	7.08	1.68	4–10	39.07	< 0.001	LR > HR-ASD* HR-ASD > HR-no***
ADI-R Algorithm sc^b^	1.77	1.74	0–5	4.95	5.36	0–17	8.44	6.31	1–18	4.97	0.020	LR < HR-no* LR < HR-ASD*
VABSsoc Standard sc	99.36	7.73	81–114	91.78	10.96	68–108	82.92	7.28	66–90	10.03	< 0.001	HR-ASD < LR*** HR-ASD < HR-no* LR < HR-no†

*LR* Low-risk siblings, *HR-no* high-risk siblings with no ASD, *HR-ASD* high-risk siblings with ASD, *ASD* Autism Spectrum Disorder, *Mullen VIQ* Mullen Scales of Early Learning Verbal IQ, *Mullen NVIQ* Mullen Scales of Early Learning Non-verbal IQ, *ns* nonsignificant, *SpecVoc* specific vocabulary, *VABSsoc* Vineland Adaptive Behavior Scales-II socialization subscale. *F F* or Brown-Forsyth F*(Age. SpecVoc and ADI-R); *Post hoc* Bonferroni or Games-Howell, *GC* gestalt closure, *sc* scores, *ADOS-2* Autism Diagnostic Observation Schedule-2, *ADI-R* Autism Diagnostic Interview-Revised

^a^LR = *n* 14; HR-noASD = *n* 22; HR-ASD = *n* 11

^b^LR = *n* 13; HR-noASD = *n* 22; HR-ASD = n 9

†*p* < .10; *p < .05; **p < .01; ***p < .001

### Measures

We used tasks in accordance with the CHC definitions of local and global performance (“flexibility of closure” and “closure speed”: Flanagan and Dixon 2013, p. 8). Measures and procedures are presented in a more comprehensive way in the supplementary material (Online resource 1).

#### Local Measures

In this modified version of the *Children´s Embedded Figures Test* (CEFT; Karp and Konstadt 1963) six pictures and the triangle-shape were used. The child was first asked to detect the triangle shape in the picture, and was given a cut-out triangle model to search for the triangle after 10 s, if not finding it spontaneously. Total exposure time for each picture was 30 s. Spontaneous pointing at the triangle was given 2 points and detection with the model-triangle was given 1 point (max. 12). If the child did not find the target within total exposure time, 0 points were given. Response latency was measured from the picture being visible to the participant and to him or her pointing spontaneously at the triangle. Due to variations in planning performance of the participants moving the triangle, no exact response latency was calculated for the model triangle condition. Finding the target with the model was counted as 20 s and not finding the triangle was counted as 30 s, the latter being maximum exposure time.

In the *Figure Ground task* (FG; Roid and Miller 1997) the child was asked to point to a part or a detail in each of 14 realistic coloured complex pictures. Exposure time was 20 s for each picture. For accuracy, 1 point was given for pointing at the target within 20 s and 0 points when failing to detect the target. (max. 14). Response latency was measured for the five most frequently answered items from the picture being visible to the participant to the pointing at the target. If the participant failed to detect the target, the response latency was set to maximum exposure time, i.e., 20 s.

In *Hidden Pictures* (HP; Roid and Sampers 2004) seven slightly differently depicted stars in the first picture and nine X-shapes in the other, are hidden in coloured realistic sceneries. Participants were asked to find and point at as many targets as possible. For the X-shape task, the target was pointed out after 10 s if the child had not found any X-shapes. Time limit was 30 s for finding stars and 45 for finding X-shapes. Accuracy was measured as the total amount of correct targets for stars and X-shapes (max. 16). Response latency was measured from the picture being visible to the participant to the pointing at the first star and X, respectively. When pointing out the target after 10 s, the latency was measured as 10 s plus the time lapse from the administrator pointing at the X to the participant pointing at another X. If the participant failed to find the targets, the latencies were set at total exposure times, i.e., 30 s for the star task and 45 s for the X-shape task.

#### Global Measures

In the *The Fragmented Picture Test* (FPT; Kessler et al. 1993; Snodgrass and Vanderwart 1980) the participant is exposed to a fragmented animate or object that gets more and more complete and the threshold is set to the picture were the object is correctly named. In this modified version four levels (towards completion) retouched into seven levels, were used. The tasks were presented in Windows 2010 Power Point-format on a lap-top computer screen. Each picture-sequence was pre-programmed to be shown for 4 s. Accuracy was the sequence of correct detection. One point was assigned to the complete picture and seven to the most fragmented. No or wrong answers were given 0 points (max. 28). This measure reflects both accuracy and response latency as each sequence was set at 4 s. However, results are reported as accuracy scores in the analyses.

In *Gestalt Closure* (GC; Kaufman and Kaufman 2004) the child is supposed to name an inkblot drawing representing fragmented objects or animates. The test was administered according to the manual with the stopping rule of ending the test after four successive failures. Accuracy was the sum of correct answers, 1 point for each correct answer (max. 24). Response latency was based on the six most frequently answered items (butterfly, clock, flower, face, dog, bed), calculated from the picture being visible to the child, to the first syllable of the correct naming of the target. At least three items had to be correctly answered for the calculation of mean. Maximum exposure time was 15 s.

### Procedure

All tasks were administered on one occasion in a clinical lab setting lasting about 20 min with a parent present. The order of the tasks was administered according to Latin square counterbalancing. Sessions were video-recorded for off line analyses using an XProtect Smart Client video system. For the global GC test Adobe Premiere Pro SC5 software was used for off-line auditory and visual analyses. Time resolution was 25 frames per second (i.e. each frame lasts 40 ms). Response latencies were manually coded and stored in an excel file (in seconds).

### Analyses

Statistics were performed in SPSS 24 (IBMCorp 2016). Statistical analyses were conducted variable-wise as the distribution of missing values varied among tasks due to missing recordings or participants looking away or being distracted, compromising analyses of latency or accuracy scores. The number of participants in each task is presented in Table 2. According to Shapiro Wilk testing, 3 out of 27 variables were not normally distributed (global GC, local HP accuracy, HP latency in the HR-noASD group). Considering the majority of data being normally distributed and analysis of variance (ANOVA) being robust against moderate violations of normality (Lix et al. 1996), parametric inference statistics were chosen. One-way ANOVAs were calculated for group comparisons. Moreover, analyses of covariance (ANCOVA) were conducted, controlling for NVIQ. A two-tailed alpha-level of 5% was applied for significance. Homogeneity was violated for accuracy and latency on HP and were analysed by Brown and Forsyth´s F*. All other variables were analysed by *F*. Post hoc were conducted with Bonferroni (when *F-value*) or Games-Howell tests (When Brown and Forsyth´s F*), the latter given different sample sizes. Effect-sizes for significant results were calculated with Cohen´s *d*, using pooled standard deviations (*s*_*p*_) given the difference in sample size, and partial eta squared (partial *η*^*2*^) for the *ANCOVA* result. Corrections for multiple comparisons across the one-way *ANOVAs* were conducted by the method of False discovery rate (Benjamini and Hochberg 1995). Apart from this correction across the *ANOVAs*, the Bonferroni/Games Howell *p* values were also corrected for multiple comparisons by false discovery rate, when the main result between groups was significant. Post-hoc power analysis (G-power, *F*-test, post-hoc *ANOVA* one way) showed that this study had acceptable power to detect large effects (1 − β = 0.64), but not medium or small effects (1 − β = 0.01–0.56).

**Table 2:** Group comparison for global and local tasks

	LR				HR-noASD				HR-ASD				Group comparisons					
	*n*	*Mean*	*SD*	*Min–Max*	*n*	*Mean*	*SD*	*Min–Max*	*n*	*Mean*	*SD*	*Min–Max*	*F*/F*	*p* ^*a*^	Group	Post hoc *P* ^*b*^		Cohen´s *d*
FPT acc	11	14.27	3.55	9–22	20	16.55	3.02	12–24	12	15.83	3.16	10–20	1.80	0.401	1 vs. 2	0.195		− 0.69
															2 vs. 3	> 0.99		0.23
															1 vs. 3	0.747		− 0.46
GC acc	14	6.64	2.41	4–12	21	8.10	3.56	2–14	11	8.36	2.54	5–12	1.30	0.483	1 vs. 2	0.514		− 0.48
															2 vs. 3	> 0.99		− 0.09
															1 vs. 3	0.496		− 0.70
GC lat	14	1.88	0.52	1.15–3.20	18	1.81	0.49	1.32–3.10	11	1.75	0.28	1.28–2.18	0.28	0.756	1 vs. 2	> 0.99		0.15
															2 vs. 3	> 0.99		0.16
															1 vs. 3	> 0.99		0.33
CEFT acc	12	4.92	2.43	2–9	21	4.05	2.25	0–8	10	4.50	2.99	0–9	0.48	0.700	1 vs. 2	> 0.99		0.37
															2 vs. 3	> 0.99		− 0.17
															1 vs. 3	> 0.99		0.15
CEFT lat	12	21.03	4.55	12.81–26.49	20	22.51	4.35	14.21-30.00	9	19.93	5.79	10.89–28.33	1.00	0.483	1 vs. 2	> 0.99		− 0.33
															2 vs. 3	0.552		0.50
															1 vs. 3	> 0.099		0.21
FG acc	14	5.64	2.98	2–11	21	7.05	2.99	3–12	10	6.40	2.32	3–10	1.02	0.483	1 vs. 2	0.484		− 0.47
															2 vs. 3	> 0.99		0.24
															1 vs. 3	> 0.99		− 0.28
FG lat	14	11.71	4.61	4.78–18.37	20	8.63	4.15	3.31–16.02	10	8.78	4.96	2.81–16.41	2.18	0.401	1 vs. 2	0.167		0.70
															2 vs. 3	> 0.99		− 0.03
															1 vs. 3	> 0.370		0.61
HP acc	13	10.38	2.10	6–14	21	10.57	3.49	5–15	9	13.89	1.54	12–16	7.34	0.018	1 vs. 2	> 0.979		− 0.06
															2 vs. 3	0.003	*HR-no < HR-ASD*	− 1.23
															1 vs. 3	0.001	*LR < HR-ASD*	− 1.90
HP lat	13	9.50	2.75	5.03–14.16	18	10.50	8.27	1.68–23.67	8	6.38	3.10	2.64–10.09	1.91	0.401	1 vs. 2	0.882		− 0.16
															2 vs. 3	0.178		0.66
															1 vs. 3	0.084		1.07

*FPT* Fragmented Picture Test, *GC* Gestalt Closure, *CEFT* Children’s Embedded Figure test, *FG* Figure Ground tests, *HP* Hidden Pictures, *acc* accuracy, *lat* latency seconds, *ASD* Autism Spectrum Disorder, *LR* Low-Risk siblings, *HR-no* High-risk siblings with no ASD, *HR-ASD* High-risk siblings with ASD

1 = LR, 2 = HR-noASD, 3 = HR-ASD, *p*^*a*^ = *p* values for the one-ways *ANOVAs* corrected for multiple comparisons, *P*^*b*^ = *p* values for Bonferroni/Games Howell post hoc tests; F* = Brown Forsyth

## Results

Mean, *SD* and range for local and global measures and results are presented in Table 2. There was a between-group effect, after correcting for multiple comparisons across the one-way *ANOVAs*, on HP accuracy (F* (2, 39.46) = 7.34, *p* = .018), with post hoc Games Howell tests showing the HR-ASD group to have higher scores than both HR-noASD (*p* = .003, *s*_*p*_ = 2.69, *d* = 1.23) and LR groups (*p* = .001, *s*_*p*_ = 1.84, *d* = 1.90). Applying correction for multiple comparisons also within the Games-Howell test for the HP- results, the results remained significant between the HR-ASD group on one hand, and the HR-noASD group (*p* = .041) and the LR group (*p* = .027), on the other. There were no group differences for accuracy and response latency for the other local measures (*F*/F* (2, 36–42) ≤ 2.18, *p* ≥ .401), nor for the global measures (*F* (2, 40–43) ≤ 1.80, *p* ≥ .401).

As non-verbal IQ differed between the LR and HR-ASD groups (see Table 1), a between group ANCOVA was conducted with HP accuracy scores as dependent variable and MSEL’s nonverbal IQ as covariate. The effect remained between groups (*F* (2, 39) = 3.99, *p* = .027, partial *η*^*2*^ = 0.170), and pairwise comparisons with Bonferroni correction showed that the HR-ASD group had higher accuracy scores (*M*^a^ = 13.68) than the LR (*M*^a^ = 10.48, *p* = .049) and HR-noASD (*M*^a^ = 10.60, *p* = .035) groups.

## Discussion

In this study we examined visual processing in early ASD testing separate measures for local and global performance in siblings with ASD (HR-ASD), siblings with no ASD (HR-noASD group) and typically developing children (LR group) and found a group difference for the local measure *HP*. Consistent with our prediction and in line with theories of enhanced visual local processing (e.g., Mottron et al. 2006), 3-year old children with ASD performed superior to the other groups. However, and against our expectations, there were no differences for global measures.

Unlike other studies (Morgan et al. 2003; Pellicano 2006), higher accuracy or shorter latencies in ASD were neither found on the CEFT nor the FG test. These tasks pose other executive demands than the HP, such as keeping attention when turning pages and processing different backgrounds, and, for the FG, also presenting different targets in each task. As executive functions are often found to be altered in individuals with ASD (Lai et al. 2017), executive control, flexibility and planning might thus have camouflaged potential superior local visual processing. It could well be that the simpler testing set-up in HP, such as looking for several targets in the same background, using only two pictures, leads to clearer differences between groups, particularly in young ages. However, talking against this, the result on the global GC did not differ between groups despite similar executive demands in the task. Unlike the other two local tasks, HP demands to find several of the same targets, different in size and angle. Superior HP performance in ASD could then either reflect that individuals with ASD show better visual form constancy (i.e., the ability to recognize similarity in form despite differences in sizes and angels) or enhanced visual search of finding several targets in a background.

Our results on global tasks are consistent with research reporting no global disadvantage related to ASD or autism symptoms (Mottron et al. 2003; Wang et al. 2007) but are inconsistent with specific fragmented picture research by Booth and Happé (2016), finding inferior global performance in ASD. Compared to the latter, our sample differed in age, gender and IQ. It is also possible that divergent trajectories of global performance might not have emerged yet in 3-year olds.

### Limitations

Our findings should be interpreted with adequate caution given that they are based on small sample size, limiting generalizability and power to detect small and medium differences on local and global tasks. Nevertheless, the result on HP remained sound, even after corrections for multiple comparisons both between and within *ANOVAs*. There were also limitations concerning tasks used in this study, as some of them originally were constructed for use in older children and were here implemented in 3-year-olds for the first time. Moreover, latency measures were operationalized by only a few items, possibly limiting their accuracy, and the number of participants that completed the different tasks varied. In addition, unlike other studies, more girls than boys participated, possibly affecting results (Bölte et al. 2011; Lai et al. 2013; Kimchi et al. 2009).

## Conclusions

This study shows that enhanced local performance is evident in children with ASD already at the age of 3, reflected by superior performance on the local measure Hidden Picture, independent of general developmental level and vocabulary. Our findings suggest that the testing of visual local performance, for example by a task as the HP, could add value to the clinical characterization of children with early suspicion of ASD. Further research on larger samples of young children with ASD is needed to investigate Hidden Pictures in relation to other local measures, visual search tasks as well as in relation to performance on form constancy.

## Electronic supplementary material

Below is the link to the electronic supplementary material.

Supplementary material 1 (DOCX 42 KB)
