# Supplementary material for: Local and Global Visual Processing in 3-Year-Olds With and Without Autism
Source: J Autism Dev Disord. 2018 Feb 6;48(6):2249–57. doi: 10.1007/s10803-018-3470-8 (PMC5948270; doi:10.1007/s10803-018-3470-8)
Supplement: Supplementary file 1 — Supplementary material 1 (DOCX 42 KB) [file 10803_2018_3470_MOESM1_ESM.docx]

**Supplementary information (Online resource 1)**

Visual Processing in 3-year-olds With and Without Autism

Journal of Autism and Developmental Disorders

Elisabeth Nilsson Jobs^1,2,3^*, Terje Falck-Ytter^1,2,3^, Sven Bölte^2,3,4^

^1^ Uppsala Child and Baby Lab, Department of Psychology, Uppsala University, Uppsala, Sweden

^2^ Center of Neurodevelopmental Disorders (KIND), Division of Neuropsychiatry, Department of Women’s & Children’s Health, Karolinska Institutet, Stockholm, Sweden

^3^ Center of Psychiatry Research, Stockholm County Council, Stockholm, Sweden

^4^ Child and Adolescent Psychiatry, Stockholm County Council, Stockholm, Sweden

*corresponding author: [elisabeth.nilsson_jobs@psyk.uu.se](mailto:elisabeth.nilsson_jobs@psyk.uu.se); elisabeth.nilsson.jobs@ki.se

**Tasks and Measures**

The tasks were chosen in accordance with the CHC definitions of local and global performance ("flexibility of closure" and "closure speed": Flanagan and Dixon 2013, page 8).

**Local Measures.** The *Children´s Embedded Figures Test (CEFT)* was derived from the Embedded Figures Test. The CEFT was standardised for children aged 5 to 12 years (Karp and Konstadt 1963) and adequate reliabilities have been reported (Amador-Campos and Kirchner-Nebot 1997). The test comprises twenty-five pictures that are presented where a shape, a “house” or a “tent” are to be detected in a complex background. The Embedded Figures Test has been frequently used within ASD-research (White and Saldaña 2011) and research on autistic traits (Cribb et al. 2016) as a measure on local processing. Individuals with ASD have been found to spot the shapes faster than individuals with TD, indicative of a detailed-focused cognitive style (Happé and Frith 2006). After piloting, the test was modified. The tent was chosen as target. The house was not included as this target was found to be generally harder to detect for this age-group than the tent and using two targets would have added un-necessary complexity to the task. Furthermore, six pictures (clown, house, truck, boat, umbrella and doll) were chosen out of the 25 original pictures. The white triangle with black edges was depicted on the side of each picture. Each picture was presented one by one to the participant. A cut-out white triangle with black edges was presented to the participant as a model. An initial training part was administered to give examples of the triangle target and examples of other non-target shapes, sweeping the cut-out model over the training picture and showing the participant when the model fitted and when not. In addition, in the first item, the triangle was pointed out in the picture if the child had tried to find the target spontaneously or with the model but failed. The task began with the administrator hiding the picture by holding one hand over it, while pointing at the target on the side, instructing the child: “Look here, find one like this, where could it be, point to it” and then removing the hand, making the picture visible to the child. If the child could not find the triangle spontaneously after 10 seconds, it was given the cut-out model with the instruction to search for the triangle in the picture by moving the triangle around in the picture. Total exposure time for each picture was 30 seconds. Measures were accuracy where the spontaneous pointing at the triangle was given 2 points and the detection of the target-triangle after searching with the model-triangle was given 1 point (max. 12 points). If the child did not find the target within 30 seconds, 0 points were given. Response latency was measured from the picture being visible to the participant and to him or her pointing spontaneously at the triangle. Due to variations in planning performance of the participants moving the triangle, no exact response latency was calculated for the condition when the modelling with triangle was used. Instead, finding the target with the model was counted as 20 seconds and not finding the triangle was counted as 30 seconds, the latter being maximum exposure time.

The *Figure Ground task* is a subtest of visualization from the non-verbal Leiter International Performance Scale-Revised (Leiter-R). It assesses visual interference, where a hidden percept needs to be detected in complex backgrounds. The Figure-Ground task has demonstrated retest reliability and internal consistencies of r = .74 to .79 in 3-year olds (Roid and Miller 1997; Sullivan 1998). In the Figure-Ground task, participants are asked to find and point to a part or a detail in a realistic coloured complex picture, e.g. a tap on a wall or a hat worn by a clown. Unlike the Children´s Embedded Figures Test, targeting the same triangle, this task contains different targets for each picture. In the original version, separate cards were used, depicting the targets that should be identified. In this version the targets were depicted on the side of each picture, presented one by one to the participant. Originally, some of the targets were depicted in black and white but in this version they were all in colour to suit the age group. Fourteen pictures, including one training item, were presented. The task began with the administrator hiding each picture by holding one hand over the picture, while pointing at the target on the side, instructing the child: “Look here, find this, where could it be, point to it” and then removing the hand, making the picture visible to the child. In the training item, the administrator pointed the target out if the participant failed to find it explicitly comparing the model on the side and the target. Total exposure time was 20 seconds for each picture. Regarding accuracy, 1 point was given for pointing at the target within 20 seconds and 0 points when failing to detect the target within the given time frame (max. 14 points). Response latency was measured for the five most frequently answered items (sweater, plant, tap, brush, and tie) from the picture being visible to the participant to the pointing at the target. If the participant failed to detect the target, the response latency was set to maximum exposure time, i.e. 20 seconds.

*Hidden* *Pictures* is a subtest of the Merrill-Palmer Revised Scales of development (Roid and Sampers 2004) that has been demonstrated good to excellent reliabilities (r = .87 to .98) in children aged 25 to 48 months. It taps visual search for several of the same targets (X-shapes and stars), slightly differently depicted in a picture. The hidden targets, stars in the first picture and X-shapes in the other, are hidden in colourful realistic scenery. Participants are asked to find and point at as many of the targets in the picture as possible. In total, seven stars and nine X-shapes are hidden. The target shape was depicted on the side of each picture. The task began with the administrator hiding the picture by holding one hand over it, while pointing at the cue, saying: “Can you find any more of this” removing the hand and saying “…here”, making the picture visible to the child. A test trial was administered to clarify the task to the child (finding moons in a picture). If the participant failed to detect a hidden moon in the test trial, the administrator proceeded with the comment: “One of the moons is hidden, can you see it?” and then pointed it out if the participant failed to spot it. For the star task, no further help was given. For the X-shape task, a prompt was given after 10 seconds if the child had not found any X, i.e. the administrator pointed at a specific X in the centre of the picture saying: “I see one here, can you see any other”. Time limit was 30 seconds for finding stars and 45 for finding X-shapes. Accuracy was measured as the total amount of correct targets for stars and X-shapes (max. 16 points). Response latency was measured from the picture being visible to the participant to the pointing at the first star and X, respectively. When a prompt was necessary after 10 seconds, the latency was measured as 10 seconds plus the time lapse from the administrator pointing at the X to the participant pointing at another X. If the participant failed to find the targets, the latencies were set at total exposure times, i.e. 30 seconds for the star task and 45 seconds for the X-shape task.

**Global Measures.** *The Fragmented Picture Test* (Kessler 1993) comprises ten pictures originating from a test on implicit memory by (Snodgrass and Vanderwart 1980). Here, the participant is exposed to a fragmented animate or object that gets more and more complete and the threshold is set to the picture were the object is correctly named. Within ASD-research, this task has previously been used as a measure of global visual processing by (Scheurich et al. 2010) and visual integration by (Booth and Happé 2016) but it has previously not been applied in pre-school aged children. In this study, after piloting the task, four pictures (elephant, pig, shoe and scissors) of the original ten, were chosen. In the original version, ten levels of descending degree of fragmentation were used for each picture. In the current study, the last four levels toward completion were used from the original version to suite the age group. In order to get a wider distribution of levels, three additional levels in between the original ones were produces from retouching the four images in the Microsoft programme Paint. The final task consisted of seven levels for each picture. The tasks were presented in Windows 2010 Power Point-format on a lap-top computer screen placed 50 cm in front of the child. The pictures were sized between 6.5 to 7 cm (length) and 3 to 5 cm (width). Each picture-sequence was pre-programmed to be shown for four seconds. The participant was instructed to look at each successive image until a correct response (i.e. naming the object) could be given or the last picture sequence had been presented. When giving a wrong answer during the sequence presentation, the participant was encouraged to keep on looking at the pictures to get the opportunity to give the correct response. The test began with two training pictures with a car and a mouse where the child first was shown the most fragmented level, gradually becoming more and more complete and then fragmented again, in order to understand that the fragmented picture was actually depicting an image. The administrator asked the child what he or she could see and commented on the picture becoming more and more blurred and then clear again as the sequences proceeded. The levels of the training pictures were produced from using the artistic effects in the Word picture tool function. A blue neutral picture was presented between the presentations of the pictures to alert the child that a new picture was due. Before the trials began and the first sequence started, the child was instructed: “Now, let´s have a look, what could this be, tell me as soon as you can”. The child was continuously prompted to look at the screen to sustain attention and asked what the picture could be. The first sequence of the picture was manually started but the succeeding sequences were pre-programmed to 4 seconds. Accuracy was measured according to the number of the picture-sequences shown until a correct answer was given. Scores were coded in reverse order, score 1 being the complete picture and 7 the most fragmented, in line with the scale direction for the other accuracy measures (the higher performance, the higher scores). No or wrong answers were given 0 points (max. 28 points). This measure reflects both accuracy and response latency as each sequence was set at 4 seconds. However, results are reported as accuracy scores in the analyses.

The *Gestalt Closure* task is a subtest of the Kaufman Assessment Battery for Children, Second Edition. It has acceptable internal consistency and test-retest reliability at the age of 3 years (*r* =.76 and .70) (Kaufman and Kaufman 2004). Participants need to name an inkblot drawing representing fragmented objects or animates. Unlike having several sequences making the picture more and more complete (as in the Fragmented Picture Test), this task presented different discrete fragmented pictures that the participant should name. The test was administered according to the manual with the stopping rule of ending the test after four succeeding failures. Accepted verbal answers, rendering a score, were applied as specified in the manual. The test began with one trial (bird), the administrator asking the child what was depicted on the page and then showing and pointing to the wings, confirming what was seen. For the following pictures, the child was instructed: “Now, let´s have a look, what could this be”. The child was continuously prompted to look at the pictures to sustain attention and asked what the picture could be. Accuracy was computed as the sum of correct answers, with 1 point given for each correct answer (max. 24). Response latency was based on the six most frequently answered items (butterfly, clock, flower, face, dog, bed), from the picture being visible to the participant, to the first syllable of the correct naming of the target. At least 3 items had to be correctly answered for being considered as valid for calculation of mean. Maximum exposure time was 15 seconds.

**Procedure**

All tasks were administered at one occasion in a clinical lab setting lasting about 20 minutes. During the session, to optimize each participant´s performance, children were verbally encouraged to stay on the tasks and prompted to attend to the task when distracted. A parent sat next to the child, but was instructed not to take any active part in the child´s performance. The order of the tasks was administered according to Latin square counterbalancing. The administrator sat opposite to the child, placed the booklet or binder on the table in front of the child, turning the pages upwards and presenting the pictures turned towards the child one by one. The administrator checked the maximum time for each item by a table clock placed out of sight from the child. For the Fragmented Picture Test, presented in Power Point on a lap top, the administrator sat beside the child, controlling the display of each picture.

Sessions were video-recorded using an XProtect Smart Client video system that also enables off line analyses of the material. For the Gestalt Closure test that involves analyses of both auditory and visual information, Adobe Premiere Pro SC5 software was used for off-line analyses. Both systems have a time resolution of 25 frames per second (i.e. each frame lasts 40 milliseconds). Using this system, response latencies were manually coded and stored in a excel file (in seconds).

**References**

Amador-Campos, J. A., & Kirchner-Nebot, T. (1997). Relations of scores on Children's Embedded Figures Test with age, item difficulty and internal consistency. [Article]. *Perceptual & Motor Skills, 85*, 675-682, doi:10.2466/PMS.85.6.675-682.

Booth, R., & Happé, F. (2016). Evidence of Reduced Global Processing in Autism Spectrum Disorder. *Journal of Autism and Developmental Disorders*, doi:10.1007/s10803-016-2724-6.

Cribb, S. J., Olaithe, M., Di Lorenzo, R., Dunlop, P. D., & Maybery, M. T. (2016). Embedded Figures Test Performance in the Broader Autism Phenotype: A Meta-analysis. *Journal of Autism and Developmental Disorders, 46*(9), 2924-2939, doi:10.1007/s10803-016-2832-3.

Flanagan, D. P., & Dixon, S. G. (2013). The Cattell-Horn-Carroll Theory of Cognitive Abilities. In *Encyclopedia of Special Education*: John Wiley & Sons, Inc.

Happé, F., & Frith, U. (2006). The Weak Coherence Account: Detail-focused Cognitive Style in Autism Spectrum Disorders. [journal article]. *Journal of Autism and Developmental Disorders, 36*(1), 5-25, doi:10.1007/s10803-005-0039-0.

Karp, S. A., & Konstadt, N. L. (1963). *Manual for the Children's Embedded Figures Test*. Oxford, England: Cognitive Tests.

Kaufman, A. S., & Kaufman, N. L. (2004). Kaufman Assessment Battery for Children Second Edition. Circle Pines, MN: American Guidance Service.

Kessler, J., Schaaf, A., & Mielke, R. (1993). Der fragmentierte Bildertest [Fragmented Picture Test]. Göttingen, Germany: Hogrefe.

Roid, G., & Miller, L. (1997). Leiter International Performance Scale-Revised: Examiners Manual. Wood Dale, IL: Stoelting Co.

Roid, G., & Sampers, J. (2004). Merrill-Palmer Revised Scales of Development. Wood Dale, IL: Stoelting Co.

Scheurich, A., Fellgiebel, A., Müller, M. J., Poustka, F., & Bölte, S. (2010). Erfasst der FBT lokale visuelle Informationsverarbeitung bei Autismus-Spektrum-Störungen? [Does the Fragmented Images Test measure locally oriented visual processing in autism spectrum disorders?]. *Zeitschrift für Kinder- und Jugendpsychiatrie und Psychotherapie, 38*(2), 103-110.

Snodgrass, J. G., & Vanderwart, M. (1980). A standardized set of 260 pictures: Norms for name agreement, image agreement, familiarity, and visual complexity. *Journal of experimental psychology: Human learning and memory, 6*(2), 174-215, doi:10.1037/0278-7393.6.2.174.

Sullivan, S. A. (1998). Leiter International Performance Scale-Revised. [Article]. *Psychology in the Schools, 35*(2), 195-197, doi:10.1002/(sici)1520-6807(199804)35:2<195::aid-pits14>3.0.co;2-7.

White, S. J., & Saldaña, D. (2011). Performance of children with autism on the Embedded Figures Test: A closer look at a popular task. *Journal of Autism and Developmental Disorders, 41*(11), 1565-1572, doi:10.1007/s10803-011-1182-4.
